# Effect of cigarette smoke condensate on gene promoter methylation in human lung cells

**DOI:** 10.1186/1617-9625-12-15

**Published:** 2014-09-05

**Authors:** Lascelles Lyn-Cook, Beverly Word, Nysia George, Beverly Lyn-Cook, George Hammons

**Affiliations:** 1 HFT-100, Division of Biochemical Toxicology, National Center for Toxicological Research, Jefferson, AR 72079, USA

**Keywords:** DNA methylation, Cigarette smoke condensate, Lung cancer cells

## Abstract

**Background:**

In lung cancer, an association between tobacco smoking and promoter DNA hypermethylation has been demonstrated for several genes. However, underlying mechanisms for promoter hypermethylation in tobacco-induced cancer are yet to be fully established.

**Methods:**

Promoter methylation was evaluated in control and cigarette smoke condensate (CSC) exposed human lung cells using the Methyl-Profiler DNA Methylation PCR System. PSAE cells were exposed to 0.3 or 1.0 μg/ml CSC for 72 hours and longer term for 14 and 30 days. NL-20 cells were exposed for 30 days to 10 or 100 μg/ml CSC.

**Results:**

Promoters of several genes, including *hsa-let-7a-3, CHD1, CXCL12, PAX5, RASSF2,* and *TCF21*, were highly methylated (>90%); *hsa-let-7a-3* was affected in both cell lines and under all exposure conditions. Level of methylation tended to increase with CSC concentration and exposure duration (statistical differences were not determined). Percentage methylation of *TCF21*, which was >98% at exposures of 10 or 100 μg/ml CSC, was found to be reduced to 28% and 42%, respectively, in the presence of the dietary agent genistein.

**Conclusions:**

Using array techniques, several tumor suppressor genes in human lung cells were identified that undergo promoter hypermethylation, providing further evidence of their potential involvement in tobacco smoke-induced lung carcinogenesis and their use as potential biomarkers of harm in tobacco smoke exposure. Results from the study also demonstrated the potential of a dietary agent to exert chemopreventive activity in human tissue against tobacco smoke related diseases through modulation of DNA methylation. Additional studies are needed to confirm these findings.

## Introduction

Epidemiological studies indicate that cigarette smoking has a strong association with lung cancer [1,2]. Approximately 80-90% of lung cancers are attributable to cigarette smoking [3]. Elucidation of the underlying mechanisms of smoke-induced lung carcinogenesis can help define future strategies for early diagnosis, prognosis, treatment, and prevention of lung cancer [4] and inform the regulation of tobacco products.

In cancer biology, the best understood epigenetic mechanism refers to DNA hypermethylation of specific loci (CpG islands) in the promoter region of known and presumptive tumor suppressor genes, which is a hallmark of human tumors, in general [5], and of lung cancer, in particular [6,7]. In lung cancer, promoter DNA methylation that leads to gene silencing is a common event, with a large number of aberrantly methylated genes having been identified [6-10]. Furthermore, methylation has been described as an early event in lung tumorigenesis [7]. Currently, DNA methylation appears as one of the most promising epigenetic biomarkers, which can improve the early detection of cancer and subsequent management of patients with diagnosed malignancy.

A positive correlation between tobacco smoking and promoter hypermethylation in human lung cancer tissue has been demonstrated for several genes (reviewed in [11]). However, the underlying mechanisms for promoter hypermethylation in tobacco-induced cancer are yet to be fully established. In this study, array techniques were used to assess the promoter methylation status of a lung cancer-focused panel of genes in two noncancerous human lung cell lines exposed to cigarette smoke condensate (CSC) both short-term and longer term, in order to identify genes whose methylation status can be affected by CSC exposure, which can provide increased insight into potential molecular mechanisms and biological pathways behind the development and progression of this tobacco-related disease as well as assisting in discovering biomarkers useful for its detection and monitoring.

## Materials and methods

### Cell lines and treatment conditions

The human lung cell lines, PSAE (primary small airway epithelial) cells (PCS-301-0100), NL-20 (immortalized bronchial epithelial) cells, A549 (adenocarcinomic alveolar basal epithelial) cells and H1299 (non-small lung carcinoma) cells, were obtained from the American Type Culture Collection (ATCC) (Manassa, VA). The cells were cultured in growth medium as recommended by the supplier. Cigarette Smoke Condensate (CSC) was purchased (Murty Pharmaceuticals; Lexington, KY), which was prepared from Kentucky standard cigarettes (1R3F; University of Kentucky, Lexington, KY) using a smoking machine designed for Federal Trade Commission testing. For smoke condensate exposure experiments, cells (400,000 cells per plate) were cultured with or without CSC (in DMSO; final vol., 0.1%) for 72 hrs. Longer term exposures were for 14 and 30 days. Medium was changed every three days with new CSC for the longer term studies. At appropriate times, cells were harvested, and processed for further analysis. In experiments with genistein (Sigma-Aldrich; St. Louis, MO), the agent was also added to cell cultures exposed to CSC.

### DNA isolation and DNA methylation PCR array

Genomic DNA was extracted from cells using the QIAMP DNA Mini Kit (Qiagen Inc.; Valencia, CA) according to the manufacturer’s instructions. The methylation status of selected genes was evaluated using the Methyl-Profiler DNA Methylation PCR Array: Human Lung Cancer EpiTect Methylation Signature Panel MeAH-041 (SABioscience, Qiagen; Frederick, MD). As noted by the manufacturer, the Panel profiles the methylation status of 24 tumor suppression gene promoters whose hypermethylation has been reported in the literature the most frequently in a variety of lung tumors. Using 1.0 μg of total DNA, the analysis was conducted according to the manufacturer’s protocol. PCR was run in the iQ5 Realtime Detection System (Bio-Rad; Hercules, CA). Quantification of relative mRNA levels was carried out by determining the threshold cycle (C_T_). Cycles were programmed as specified in the Methyl-Profiler protocol.

### Methylation data analysis

The Methyl-Profiler PCR Array Excel-based data analysis template was downloaded from the SABioscience website at: http://www.sabiosciences.com/dna_methylation_analysis.php. C_T_ values were entered into the raw data table and results were automatically determined by the template through a series of mathematical equations for each gene analyzed. These equations and calculations are described in detail in the Methyl-Profiler DNA Methylation PCR Array System User Manual (SABioscience, Qiagen). Percent methylation was calculated by these equations using the appropriate C_T_ values. Heatmap analysis was performed using R statistical software (http://r-project.org).

## Results and discussion

**Figure 1:**
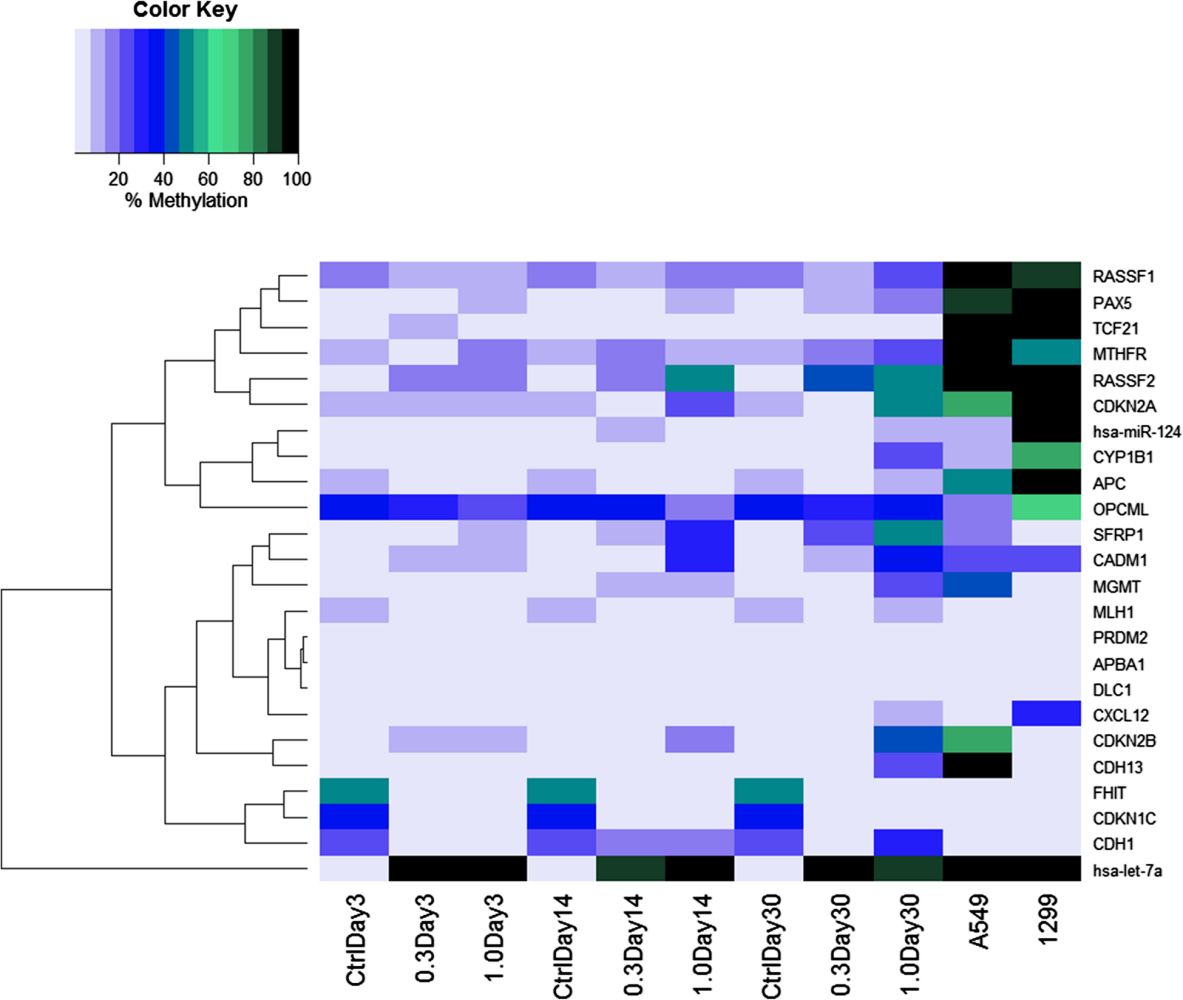
**Heatmap of the intensity of promoter methylation of the 24-genes panel in PSAE (human primary small airway epithelial) cells.** Cells were cultured in the absence or presence of 0.3 or 1.0 μg/ml CSC for 3, 14 or 30 days. Methylation was assessed by the Methyl-Profiler DNA Methylation PCR System. Methylation analysis in the lung cancer lines, A549 and H1299, is also represented. Color code and intensity indicate level of methylation.

**Figure 2:**
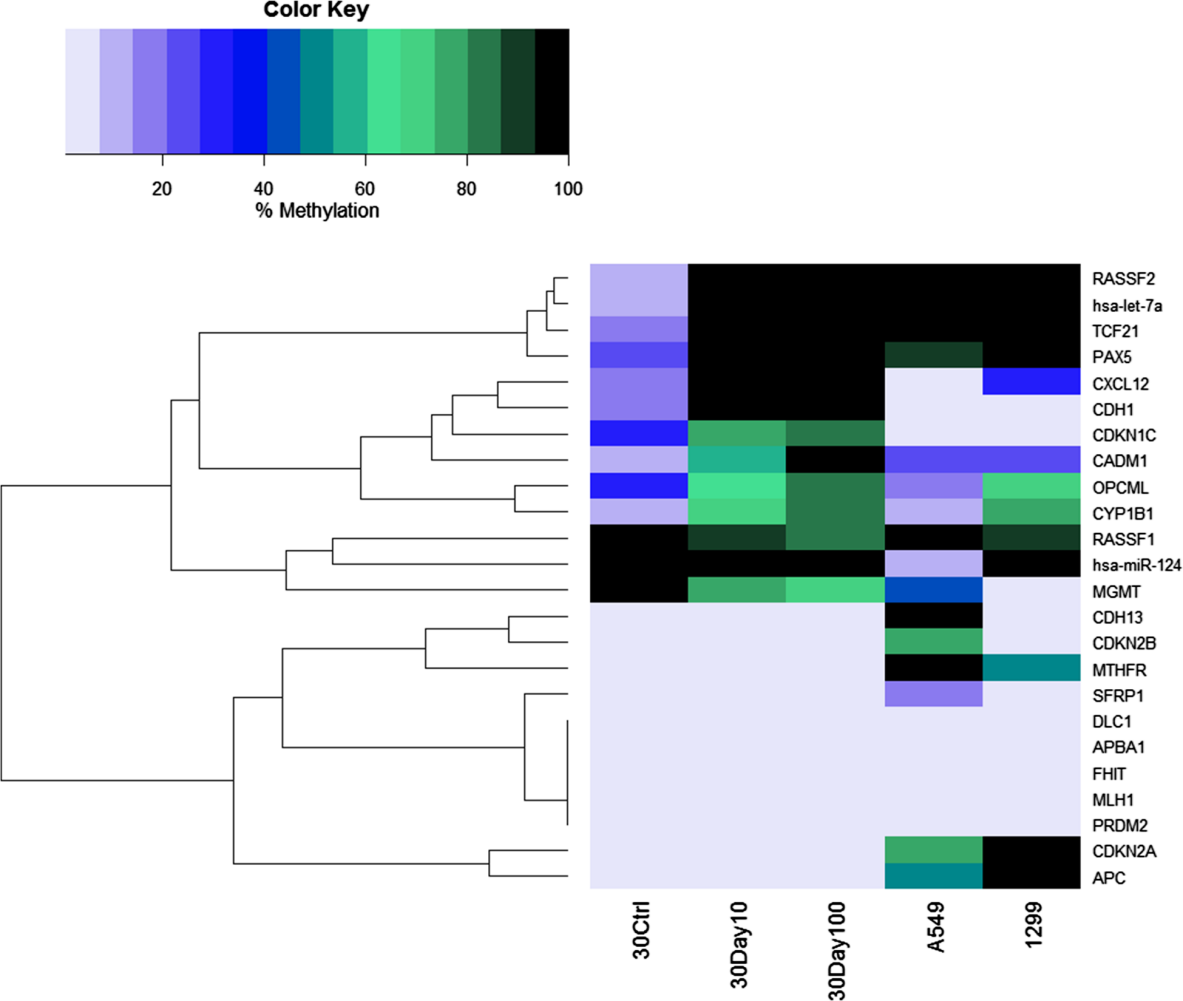
**Heatmap of the intensity of promoter methylation of the 24-genes panel in NL-20 (human immortalized bronchial epithelial) cells.** Cells were cultured in the absence or presence of 10 or 100 μg/ml CSC for 30 days. Methylation was assessed by the Methyl-Profiler DNA Methylation PCR System. Methylation analysis in the lung cancer lines, A549 and H1299, is also represented. Color code and intensity indicate level of methylation.

In the present study, the degree of promoter methylation across a defined panel of genes was evaluated in control and CSC exposed cells using the Methyl-Profiler DNA Methylation PCR System technology. The signature panel was chosen based on representation of genes that have been reported to be altered in a variety of lung cancers. PSAE cells were exposed to 0.3 or 1.0 μg/ml CSC for 72 hours and longer term for 14 and 30 days. Compared to controls (DMSO only) at these time points eight of the twenty-four genes in the panel were identified that displayed an increased percentage of promoter methylation after exposure to 1.0 μg/ml CSC for 14 or 30 days. The genes included *CDH13, CDKN2A, CDKN2B, CYP1B1, MGMT, RASSF1,* and *SFRP1* (Figure 1). Percentage methylation ranged from 19% to 53%. Increased methylation of *RASSF1* and *SFRP1* was also observed after exposure to 0.3 μg/ml CSC for 30 days. One gene, *hsa-let-7a-3,* was highly methylated (>90%) under all of the exposure conditions (Figure 1). NL-20 cells were exposed for 30 days at concentrations of 10 and 100 μg/ml CSC. At both concentrations promoters of six genes, *hsa-let-7a-3, CHD1, CXCL12, PAX5, RASSF2,* and *TCF21*, were identified as being highly methylated (>97%) (Figure 2). The percentage of methylation in four other genes, *CADM1, CDKN1C, CYP1B1,* and *OCPML*, increased with CSC concentration (Figure 2).

The gene affected in both cell lines and under all of the exposure conditions was *hsa-let-7a-3*. Let-7s are a family of highly conserved archetypical miRNAs, containing 13 members located on nine different chromosomes. Much of the available evidence supports hsa-let-7a as a lung cancer related tumor suppressor (as reviewed in [12,13]). Several cellular protein targets of *hsa-let-7a* have been identified, such as c-MYC, HMGA, STAT3, NIRF, RAS. Over-expression of *let-7a* was shown to be related to increases in overall survivals of NSCLC patients. *Hsa-let-7* is also a prognostic factor indicating the prevention of recurrence in surgically resected NSCLC patients. Expression levels of *hsa-let-7* are frequently reduced in lung cancers. Furthermore, lung cancer patients with reduced *hsa-let-7a* expression were found to have significantly worse prognosis. Hsa-let-7a inhibits growth of lung cancer cells *in vitro*. Decreased *let-7a-3* expression has also been demonstrated in breast cancer [14]. Two other studies, however, suggest that miRNA let-7a-3 may behave in favor of tumor progression [15,16]. The reasons for these contrasting results are not clear. Cigarette smoke typically induces let-7a down-regulation, as demonstrated in animal studies. miRNA let-7 has been shown to be down-regulated in the lungs of rat exposed to environmental tobacco [17], as well as in mouse lungs [18]. Although results from several studies have also shown that DNA methylation may be involved in the regulation of let-7a-3 [14-16], there have been no previous reports of promoter methylation of this gene in response to tobacco smoke exposure.

**Figure 3:**
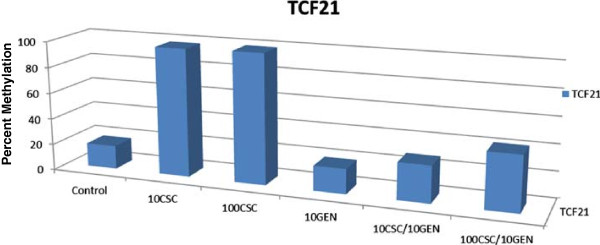
**Effect of genistein on CSC-induced methylation.** NL-20 cells were exposed to 10 or 100 μg/ml CSC for 30 days in the presence of 10 μM genistein. Cells were also treated with genistein in the absence of CSC. Methylation was assessed by the Methyl-Profiler DNA Methylation PCR System.

The ability of the dietary agent genistein to modulate CSC-induced promoter methylation was also explored. Increasing evidence indicates that an important pathway in the chemopreventive activities of dietary components is their ability to regulate the epigenome [19]. Genistein, one of the soy-derived bioactive isoflavones, modulates DNA methylation. For example, genistein was found to decrease methylation of BRCA1, GSTP1, and EPHB2 promoters in prostate cancer cell lines [20]. In the present study, NL-20 cells were exposed to CSC in the presence of genistein (10 μM). Promoter methylation was assessed in the twenty-four genes panel. Under the conditions employed, the percentage methylation of *TCF21*, which was >98% at exposures of 10 or 100 μg/ml CSC, was reduced to 28% and 42%, respectively, in the presence of genistein (Figure 3). The level of methylation in other genes was not altered. The transcription factor TCF21 is widely expressed; its normal function is to promote mesenchymal transition into epithelial cells [21]. It has been identified as a tumor suppressor gene [22]. *TCF21* is reported to be frequently hypermethylated and silenced in head and neck and lung cancers, and restoration of *TCF21* expression inhibited tumor growth, both in a lung cancer cell line and in a mouse xenograft model [22]. The results found in our study add support that up-regulation of hypermethylated tumor suppressor genes may be a potential pathway for chemoprevention in tobacco smoke-induced lung cancer and that the methylation status of the tumor suppressor gene *TCF21* may be an important biomarker for monitoring the chemopreventive activity.

Several limitations of this study are noted. CSC is widely used in model systems to study *in vitro* effects of tobacco smoke [23-26]. A disadvantage is that cell culture models often do not exhibit all the differentiated and functional characteristics of the corresponding native epithelium or the entire organ. A concern can also be the inherent instability, especially on long term culture. Treatment with CSC may not exactly replicate *in vivo* responses to smoke exposures. However, in *in vitro* research, cellular and subcellular functions can be studied with more ease in a simplified, direct biological model system using guidelines for good cell culture practice, which allows the prediction of mechanisms that may be relevant to *in vivo* situations. In the current study, two cell lines were included to permit assessing effects at various transformational stages and to meet the need to examine results in more than one cell line. The CSC dose range used, although difficult to compare with that in human airways in smokers, is similar to those previously reported in other *in vitro* studies (0 – 125 μg/ml) [23-26]. Additionally, the average yield of CSC was 26.1 mg/cigarette. Therefore, the dose of 1 μg/ml is reachable if an individual with 60 kg body weight takes only 3–4 cigarettes and all of the smoke was absorbed [27]. It is quite possible that doses up to 100 μg/ml could be reached in the human airways of smokers, especially concerning the accumulation of smoke compounds over a smoker’s life [28]. The array approach employed in the study permitted the surveying of a panel of 24 genes that included multiple time points and concentrations. However, for each time-concentration point an individual 96-well plate was required. As such, multiple determinations were not conducted; statistical analysis of significant differences in methylation levels could not be included. Validation of the array data will be needed and should be done in future studies for those genes of interest. Despite these potential limitations, our experiments yielded several interesting and potentially relevant findings.

## Conclusions

This study, using the array techniques, identified several tumor suppressor genes in human lung cells whose promoter methylation was increased upon exposure to CSC, providing further evidence of their potential involvement in tobacco smoke-induced lung carcinogenesis. The miRNA hsa-let-7a may play a critical role and provide a potential biomarker of harm in tobacco smoke exposure, given the observations from both cell lines included in the study. Results from the study also demonstrated the potential of a dietary agent to exert chemopreventive activity in human tissue against tobacco smoke related diseases through modulation of DNA methylation. Additional studies are needed to confirm these findings, to screen other genes and other cell lines, and to further characterize the role and impact of epigenetic changes in tumor suppressor genes in tobacco smoke-induced harm.

## Competing interests

The authors declare that they have no competing interests.

## Authors’ contribution

LL-C carried out the DNA isolation and DNA methylation PCR array analysis and the methylation data analysis. BW conducted the cell culturing and treatments. NG participated in the methylation data analysis. BL-C assisted in research designing, data interpretation, and drafting of the manuscript. GH directed the research design and data interpretation and drafted the manuscript. All authors read and approved the final manuscript.
